# Nitrogen and Microelements Co-Drive the Decomposition of Typical Grass Litter in the Loess Plateau, China

**DOI:** 10.3390/plants13060753

**Published:** 2024-03-07

**Authors:** Yun Xiang, Haoning Chen, Weiqi Feng, Yongli Wen, Ying Xie, Man Cheng, Hua Li

**Affiliations:** 1 College of Resources and Environment, Shanxi Agricultural University, Jinzhong 030801, China; xiangyun@sxau.edu.cn; 2 Institute of Loess Plateau, School of Environmental & Resource Sciences, Shanxi University, Taiyuan 030006, China; chn13753900846@163.com (H.C.); 202323202006@email.sxu.edu.cn (W.F.); ylwen@sxu.edu.cn (Y.W.); 3 Shanxi Dadi Minji Ecological Environment Company Limited, Taiyuan 030002, China; xieying19926@163.com

**Keywords:** litter mixture, litter mass remaining, microelement, non-additive effect

## Abstract

In grassland ecosystems, the decomposition of litter serves as a vital conduit for nutrient transfer between plants and soil. The aim of this study was to depict the dynamic process of grass litter decomposition and explore its major driver. Three typical grasses [*Stipa bungeana* Trin (St. B), *Artemisia sacrorun* Ledeb (Ar. S), and *Thymus mongolicus* Ronniger (Th. M)] were selected for long-term litter decomposition. Experiments were conducted using three single litters, namely, St. B, Ar. S, and Th. M, and four different compositions of mixed litter: ML1 (55% St. B and 45% Th. M), ML2 (55% St. B and 45% Ar. S), ML3 (75% St. B and 25% Th. M), and ML4 (75% St. B and 25% Ar. S). The dynamic patterns of mass and microelements (Ca, Mg, Fe, Mn, Cu, and Zn) within different litter groups were analyzed. Our findings indicated that, after 1035 days of decomposition, the proportion of residual mass for the single litters was as follows: Th. M (60.6%) > St. B (47.3%) > Ar. S (44.3%), and for the mixed groups it was ML1 (48.0%) > ML3 (41.6%) > ML2 (40.9) > ML4 (38.4%). Mixed cultivation of the different litter groups accelerated the decomposition process, indicating that the mixture of litters had a synergistic effect on litter decomposition. The microelements of the litter exhibited an initial short-term increase followed by long-term decay. After 1035 days of decomposition, the microelements released from the litter were, in descending order, Mg > Ca > Fe > Cu > Mn > Zn. Compared to the separately decomposed St. B litter, mixing led to an inhibition of the release of Ca (antagonistic effect), while it promoted the release of Mg, Cu, and Zn (synergistic effect). For the single litter, the stepwise regression analysis showed that Ca was the dominant factor determining early litter decomposition. Mg, Mn, and Cu were the dominant factors regulating later litter decomposition. For the mixed litter groups, Ca, Mn, and Mg were the dominant factors closely related to early decomposition, and TN emerged as a key factor regulating the mass loss of mixtures during later decomposition. In summary, nitrogen and microelements co-drive the decomposition of typical grass litter. Our study underscores that, in the succession process of grassland, the presence of multiple co-existing species led to a faster loss of plant-derived materials (litter mass and internal elements), which was primarily modulated by species identity and uniformity.

## 1. Introduction

Grasslands play an important role in carbon sequestration, water conservation, maintaining biodiversity, etc., in the terrestrial ecosystem. In grassland ecosystems, the decomposition of litter serves as a vital conduit for nutrient transfer between plants and soil [1]. This interconnection plays a pivotal role in preserving soil fertility, fostering plant reproduction, and bolstering ecosystem resilience. Litter undergoes decomposition through physical, chemical, and biological pathways, releasing energy and nutrients or transitioning into various ecosystem components in a stable form [1,2,3,4]. This process facilitates plant reproduction and soil carbon sequestration [5]. The decomposition layer typically comprises litter from multiple plant species sources [6], and the neighborhood effects generated by these mixtures are significant and thus cannot be overlooked [7]. Litter mixing is anticipated to either inhibit (i.e., have an antagonistic effect on) or promote (i.e., have a synergistic effect on) decomposition [6,8,9]. This is a non-additive effect that may change throughout the process of decomposition [10,11]. Although there is no consensus, these effects can be attributed to the characteristics, uniformity, diversity, richness, and chemical structure of the species in the mixture during the decomposition process [11,12,13,14,15,16].

The interactions between mixed litters during the decomposition process can significantly alter the cycling flux and energy and nutrient patterns in ecosystems [13,17,18,19]. Some species may exert harmful or beneficial effects on other species or microorganisms by secreting decomposition products into the environment [20,21,22]. Litter that decomposes slowly can impede the decomposition of adjacent litter by releasing inhibitory compounds such as phenolics [23]. Conversely, the migration of N and P from high-quality litter to low-quality litter may facilitate decomposition [16]. Therefore, it is imperative to clarify the impact of the mixed effects of litter on the material cycling capacity of soil and plant ecosystems. However, there is a dearth of research focusing on the decomposition and elemental release characteristics of mixed litter in grassland ecosystems in the ecological restoration area of the Loess Plateau. This gap may hinder our understanding of the interrelationship among different components in ecologically fragile areas, obstruct the assessment of energy and nutrient balance, and limit the application of ecological restoration.

In addition to macroelements such as nitrogen (N) and phosphorus (P) that drive litter decomposition, the role of microelements has been established as indispensable [24,25,26,27]. Microelements, such as manganese (Mn), are widely acknowledged as important factors in lignin degradation, which is actively involved in promoting litter decomposition [28]. Calcium (Ca) and magnesium (Mg) have been identified as reliable predictors of litter decomposition rate and mass loss rate [25,27]. Iron (Fe) can be released into the soil and form resilient mineral-associated complexes by combining its oxidized state with plant-derived organic matter, thereby slowing down decomposition [29]. However, unlike the greater demand for macroelements and reabsorption efficiency in plants, microelements appear to be more immobilized [30]. This differential mobility between microelements and macroelements can result in the accumulation of microelement concentrations (Fe, Zn, Mn, Pb, and Cd) to excessive levels, potentially inhibiting litter decomposition [31]. Currently, there is a lack of consensus across numerous studies regarding the concentration and residual dynamics of microelements during this process, which involves the influence of species characteristics and regional conditions. Meanwhile, distinct release patterns of microelements have been observed during mixed litter decomposition compared to individual litter decomposition [19,31]. Despite the above, there remains a significant knowledge gap in this area. There is considerable potential for further exploration of the decomposition dynamics and effects of litter mixing on the release pattern of microelements. Such research could not only help identify and provide a foundation for elucidating the decomposition mechanism of litter but could also facilitate a more comprehensive understanding of nutrient cycling characteristics in grassland ecosystems.

The restoration and reconstruction of grasslands are pivotal strategies for ecological restoration in the Loess Plateau. Studying the decomposition characteristics of grassland litter holds significant ecological implications for species preservation and soil cultivation in fragile ecosystems. In this study, we established seven sets of independent litter decomposition experiments, including three sets composed of three different individual plant litters, and four sets of experimental samples formed by mixing plant litters together to simulate potential species assembly and decay dynamics during natural grassland succession in the Loess Plateau.

The objectives of our study are the following: i. investigate the impact of mixtures on the decomposition rate and how it differs from that of single litter; ii. depict the behavior of microelements during the litter decomposition process and elucidate the biogeochemical cycling process of microelements in grassland succession; and iii. clarify the key drivers of litter decomposition.

## 2. Results

### 2.1. Variation in the Mass Remaining Proportion of Different Grass Litter

**Figure 1:**
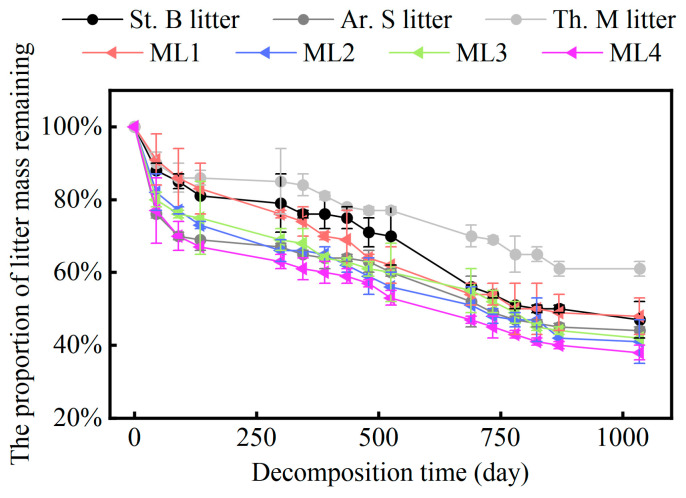
The variation in the proportion of litter mass remaining during the decomposition.

The variability in the proportion of the remaining litter mass during the simulated decomposition experiments is depicted in Figure 1. The decomposition process of both single and mixed litters followed an alternating “fast–slow” pattern, leading to a continuous decrease in residual mass. Specifically, the degradation of the St. B, Ar. S, and Th. M litters primarily occurred in four stages: fast decomposition (0–135 days), slow decomposition (136–525 days), rapid secondary decomposition (526–870 days), and stable decomposition (871–1035 days). After 1035 days of decomposition, the proportion of residual mass was as follows: Th. M (60.6%) >ML1 (48.0%) > St. B (47.3%) > Ar. S (44.3%) > ML3 (41.6%) > ML2 (40.9%) > ML4 (38.4%).

Furthermore, the Olson model was used to explore the exponential relationship between the residual proportion and cultivation time. The decomposition constant (k) represented the decomposition rate. The decomposition rates of Ar. S litter and St. B litter alone were higher than that of Th. M litter. The decomposition rate of the Ar. S and St. B mixed litter (ML2 and ML4) was higher than that of the St. B and Th. M mixed litter (ML1 and ML3) at the same mixing ratio (Table 1). We also found that the decomposition rates of mixed groups (ML1, ML2, ML3, and ML4) were higher than those of single groups (St. B, Ar. S, and Th. M). This suggested that litter mixing accelerated the decomposition process.

### 2.2. Variation in Microelements of Different Grass Litters

**Figure 2:**
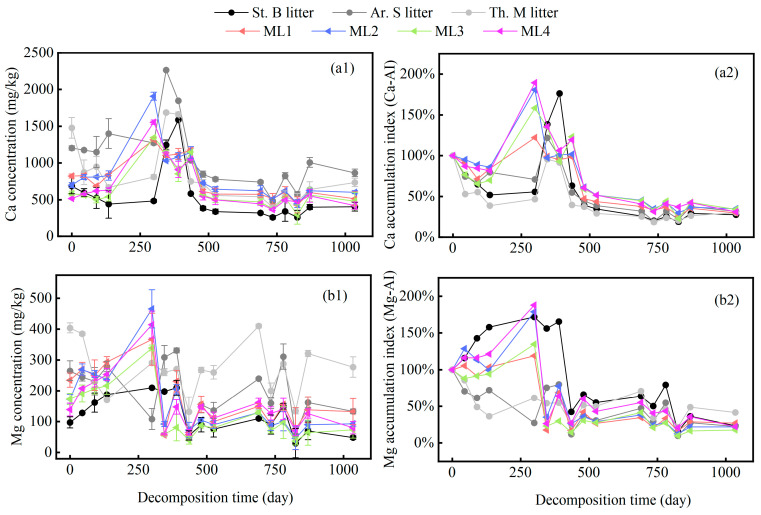
The variation in concentrations and accumulation indices of total Ca (**a1**,**a2**) and Mg (**b1**,**b2**) during litter decomposition.

In the decomposition experiment, the overall variation in the concentration of Ca and Mg in both the single and mixed litters followed a phased fluctuation pattern (Figure 2(a1,b1)). We observed a rapid increase in the concentration of Ca and Mg in the litter from 0 to 300 days, followed by a rapid decrease over approximately 435 days, and, finally, a period of fluctuating degradation until the end of cultivation. To quantify the enrichment or release characteristics of the microelements, we further calculated the litter accumulation index (AI) at each decomposition stage (Figure 2(a2,b2)). The results revealed an alternating “release–enrichment–release” pattern in the total amount of Ca and Mg in both single and mixed litters. After 1035 days of decomposition, the amounts of Ca released in each of the three single litters (St. B. Ar. S, and Th. M) and four mixed litters (ML1, ML2, ML3, and ML4) were 72.2%, 68.1%, 69.9%, 70.0%, 64.9%, 65.8%, and 68.7%. In contrast, Mg was released at rates of 76.6%, 77.7%, 58.4%, 73.0%, 78.0%, 82.4%, and 77.8%. These findings indicated that litter mixing inhibited the release of Ca (antagonistic effect) while promoting the release of Mg (synergistic effect).

**Figure 3:**
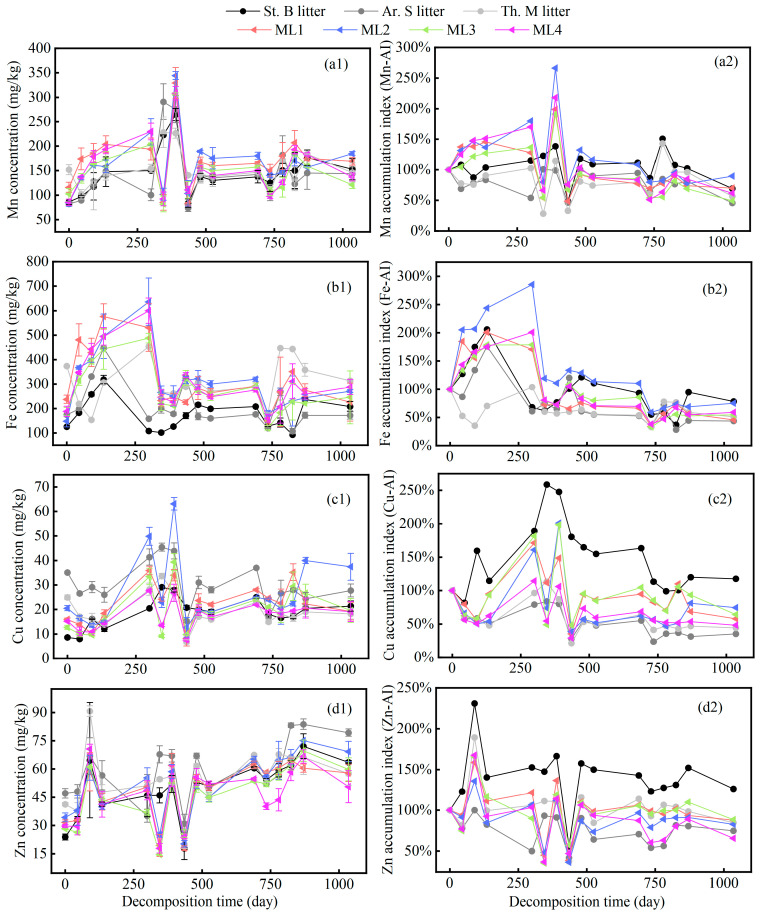
The variation in concentrations and accumulation indices of total Mn (**a1**,**a2**), Fe (**b1**,**b2**), Cu (**c1**,**c2**), and Zn (**d1**,**d2**) during litter decomposition.

The variations in the concentrations of the microelements (Mn, Fe, Cu, and Zn) in the litter are depicted in Figure 3. In contrast to Ca and Mg, the concentrations of Mn, Fe, Cu, and Zn in both single and mixed litters progressively increased in a fluctuating manner throughout the entire decomposition process. After 1035 days of decomposition, the remaining rates of Mn, Fe, Cu, and Zn were 70.1%, 58.2%, 63.1%, and 87.1%, respectively. Notably, the remaining rates of Mn and Fe in St. B and ML2 were the highest among the individual and mixed litter groups. In addition, we found an absolute enrichment of Cu (Figure 3(c2)) and Zn (Figure 3(d2)) in the St. B litter.

### 2.3. Non-Additive Effect of Litter Mixing on the Mass Remaining Rate and Element Accumulation

As indicated in Table 2, the mixing of various litters exhibited a negative non-additive effect on the mass remaining rate, indicating that the combination of different chemical litters expedited the litter decomposition process. Overall, ML4 exhibited the greatest negative non-additive effect on litter mass retention, followed by ML3. The release dynamics of Ca and Mg displayed distinct responses to litter mixing. The release proportion of Ca in mixed litter was generally slower than anticipated, whereas the observed release proportion of Mg was initially lower than expected but gradually accelerated and exceeded expectations. The observed release of Fe and Mn progressively surpassed expectations as the decomposition of the litter progressed. In other words, the effect of mixing on the release of Fe and Mn transitioned from inhibition to facilitation. We also observed negative non-additive (synergistic) effects of different mixed litters on the release of Cu and Zn. These findings suggested that litter mixing indeed altered the characteristics of element turnover.

### 2.4. Relationship between the Mass Remaining Rate and Litter Chemical Elements

The Pearson correlation analysis results revealed a significant negative correlation between the remaining mass of litter at the decomposition endpoint (1035th day) and the initial total nitrogen (TN) concentration, and a significant positive correlation with the initial lignin content (Table 3).

Based on the release characteristics of lignin and microelements, we segmented the litter decomposition into two stages: early rapid decomposition (0–525 days) and late slow decomposition (526–1035 days). We conducted a stepwise regression analysis to assess the relationships between the variations in litter lignin and microelement concentrations and the mass remaining rate during these two stages (Table 4). For individual litter decomposition treatments, the Ca concentration was the dominant factor during early decomposition, while TN, Mn, and Cu concentrations were the dominant factors during later decomposition. For mixed litter treatments, the mass loss was primarily associated with the concentrations of Ca, Mn, and Mg during early decomposition. As the decomposition process advanced, the TN concentration emerged as a key factor regulating the mass loss of the mixtures.

## 3. Discussion

### 3.1. Mixing of Different Grass Litters Promotes Decomposition

Given the prevailing climatic conditions, litter quality is recognized as a crucial factor influencing the mass remaining rate and the decomposition limit value [3,10]. In our study, after 1035 days of decomposition, the mass remaining rates of the three single litters (St. B, Ar. S, and Th. M litters) were approximately between 44.3% and 61.6%. Among these, an obviously higher mass remaining rate and slower decomposition rate were observed for the Th. M litter compared with the St. B and Ar. S litters at each decomposition. This suggests that the Th. M litter is a slow-decomposing species, while the St. B and Ar. S litters are likely fast-decomposing species. As previously discovered [7], compared to slow-decomposition species, fast-decomposition species possess lower initial lignin content and higher N concentration, which aligns with our findings (Table S1). Berg proposed that complex lignin/lignified matter is an important factor that theoretically regulates the stability and decomposition limit values of litter, although this has not been confirmed [3,26]. Recently, Walela et al. [32] provided circumstantial evidence for the regulation of limit mass retention by initial lignin/N ratio and lignin content, emphasizing the inhibitory effect of slow delayed lignin (acid-unhydrolyzable residue) on litter decomposition, resulting in more C from native woodland litters with higher initial lignin content remaining at the end of decomposition. In this study, compared to the St. B and Ar. S litters, we observed a significantly lower initial TN concentration and a higher lignin content and lignin/N ratio in the Th. M litter (*p* < 0.05), which may comprehensively explain the higher final mass remaining proportion of the Th. M litter.

In natural ecosystems, nutrient migration between litters of varying chemical compositions is considered a fundamental principle underlying litter decomposition. This concept, summarized as non-additive effects [6], has been proven to be influenced by the types and diversities of mixed litters [7,19,33]. Our study revealed different effects of specific litter mixtures on the degree of decomposition. Notably, the mass remaining rates of ML4 and ML2 were lower than those of ML3 and ML1. This finding indicated that mixtures with “fast–fast” species were more effective in synergistically enhancing plant residue degradation compared to mixtures with “fast–slow” species. This differs from the discovery made by Cuchietti et al. [7]. Our result is primarily associated with the impact of the mixture quality on the development of microbial communities. The lower content of molecular-complex lignin in St. B and Ar. S litters undoubtedly lowers the metabolic cost of microorganisms [34], thereby reducing energy consumption during the decomposition process. In addition, the role of microbial communities in the complementary utilization of resources is considered pivotal [8]. The high bioavailability of the St. B and Ar. S litters can alleviate the survival pressure on surrounding decomposer groups, provide more abundant niche space, and facilitate the development of decomposer biomass and diversity. This, in turn, promotes the synergistic and complementary metabolic functions of decomposer groups in degrading organic matter [35].

The synergistic interaction between species identity and composition patterns within communities plays a significant role in driving the degradation capacity of plant-derived organic matter in natural grassland ecosystems. As expected, the mixing ratio emerges as a significant factor in regulating the degree of decomposition under conditions of specific species composition. In this study, the litter groups with a higher proportion of St. B litter were used to simulate the later succession of grassland. We found that the mass remaining rates of ML1 and ML2, which had a lower proportion of St. B, were higher compared to ML3 and ML4, which were mixed with the same species but contained a higher proportion of St. B litter. This suggests that the litter decomposition rate increases with grassland succession, indicating that there is more material feedback during later grassland succession.

### 3.2. Release or Enrichment of Microelements during Grass Litter Decomposition

The process of releasing microelements from litter is an important pathway for the return of plants to the soil in grassland ecosystems. We observed an increase in Ca, Mg, Fe, Mn, Cu, and Zn concentrations during the early decomposition of both single and mixed litter samples. This was subsequently followed by a decrease, ultimately falling below the initial concentration. Previous studies have reported similar increase–decrease phases for the microelements of litter decomposition [36,37]. These findings suggested that the enrichment–release pattern of microelements may be prevalent in the decomposition process of litter in natural systems.

The dynamic regulation of Ca and Mg concentrations during the litter decomposition process is controlled by the same underlying mechanism [38]. Interestingly, in our study, we found that the patterns of Ca and Mg release responded differently to litter mixing. We discovered that litter mixing exerted an antagonistic effect on the release of Ca. Conversely, a synergistic effect was observed in the release of Mg. As decomposition progressed, the synergistic effect intensified, especially under the ML2, ML3, and ML4 treatments (Table 2). The impact of litter mixing on Mg release may occur in conjunction with overall mass loss, as we observed similar non-additive dynamic trends in mass retention rate (Table 2). Previous studies have emphasized that in the later stages of induction, the synergistic effect becomes stronger due to the improvement in litter quality, the increase in diversity, and the abundance of decomposers [14,39,40]. Therefore, we speculated that in our study, as the decomposition progressed, the mass loss of the mixture resulting from the synergistic effect accelerated the release of Mg more than expected. This may improve the energy acquisition and community development of microorganisms at the litter and soil interaction interface, and further provide positive feedback to the mass loss. 

Contrary to the variation in Ca and Mg concentrations, the concentrations of Fe, Mn, Cu, and Zn were generally higher than those at the initial decomposition stage after 1035 days of litter decomposition. We calculated the average mass remaining rates of Fe, Mn, Cu, and Zn, which were 50.2%, 70.1%, 63.1%, and 87.1%, respectively. This revealed that Fe was a microelement with mobility, second only to Ca and Mg. This finding differs from previous studies [36,38]. This phenomenon may be related to the formation of mineral complexes through the reaction between Fe and lignin [39,41,42,43]. In our study, we observed a rapid decrease in lignin, which could result in a proportional loss of Fe. Alternatively, a periodic hypoxic microenvironment was induced during litter decomposition, which may disrupt the stable structure between Fe oxides and lignin, thereby accelerating Fe release [29].

It is worth noting that after 1035 days of decomposition, the accumulative indices of Cu and Zn in the four mixed litters were significantly lower than that of St. B litter, indicating more absolute release (Figure 3). Further calculations revealed that the release of Cu and Zn from the four mixed litters exhibited a synergistic effect (Table 2). These results indicate that litter mixing can significantly influence the dynamic behavior of Cu and Zn in litter [22]. In summary, our findings showed that during the grassland succession process, communities consisting of multiple species actually accelerate litter decomposition and promote the release of microelements (especially Mg, Cu, and Zn). Our findings may contribute to the rational management of grassland ecosystem restoration on the Loess Plateau.

### 3.3. Correlation Analysis Reveals Important Factors Regulating Litter Decomposition

As previously reported [32,44], we found a significant positive correlation between the initial lignin content and the mass remaining rate of litter (Table 3). However, we observed a significant negative correlation between the initial N concentration in the litter and the mass remaining rate, which diverges from previous findings [3,26]. These studies argued that a higher initial N concentration could reduce the limit value of organic matter decomposition or delay reaching the limit.

The decomposition of litter aligns with its internal elemental dynamics in terms of rate [15,30]. The decomposition process of litter can be segmented into two stages: early rapid decomposition and late slow decomposition. In our study, based on the trends of lignin, Ca, Mg, Fe, Mn, Zn, and Cu in the litter, we divided the decomposition into two stages: intense fluctuation release from 0 to 525 days and slow release/stability from 690 to 1035 days. We found that the Ca, Mn, and Mg concentrations were the primary factors correlating with the mass loss of the mixed litter groups during the early stage of litter decomposition, and the Ca concentration affected the mass loss of the single litter groups. These findings suggest that the fast decomposition of mixed litter in the early stage may be associated with the cooperative interaction of multiple microelements. The Ca in the litter can contribute to the development of white rot fungus communities [45], while Mn can be used for the production of manganese peroxidase (MnP) and subsequent lignin degradation [46]. In addition, the regression analysis in our study showed a significant linear relationship between the concentrations of Ca and Mn and the lignin accumulation index in the early stages of mixed litter decomposition (Figure S1). This lignin accumulation index can effectively explain the changes in the litter mass remaining rate (Figure S2). We therefore speculate that litter mixing may result in multiple microelements synergistically contributing to the lignin decomposition process due to neighborhood effects. This could promote a faster decomposition of litter mixtures in the early stages. 

As the decomposition progressed, TN emerged as the primary factor influencing the mass loss of both single and mixed litters (Table 4), a finding that aligns with the discovery made by Berg et al. [47]. In the later stage of decomposition, the increasing TN concentration (Figure S3) may promote the mass loss of litter by forming covalent bonding with macromolecules during humification processes [48]. In addition, we identified a cooperative impact of multiple elements (TN, Mn, and Cu) on the decomposition of single litter in the late stage (Table 4), potentially leading to a delayed mass loss compared to mixed litters. From these results, we can extract two main insights: (1) Regardless of whether a single or mixed litter is used, the internal elements exhibit a clear-phased regulation of mass loss during the decomposition process. (2) The regulatory matrix for the decomposition of mixed litter differs from that of single litter. Therefore, in natural ecosystems with mixed species, especially in the Loess Plateau region, the study of litter decomposition should theoretically extend to the level of plant communities. This approach could enable more accurate control of the biogeochemical characteristics of regional material turnover. Regrettably, we only modeled the plant composition of grasslands during natural successional processes in the same site, which may be somewhat different from the actual decomposition conditions in situ [6]. Furthermore, the most vibrant biological components (e.g., phyllosphere–soil microbes and extracellular enzyme activity parameters) were neglected in our study. These have been proven to be very important in driving leaf decomposition and elemental release [49,50,51]. Regardless, our results could provide technical support for effectively regulating the process of litter decomposition in grassland, i.e., adding nitrogen or microelements could influence the decomposition rate of grass litter. This is of great practical significance for the maintenance and improvement of multiple functions such as carbon sequestration, nutrient cycling, and water retention in grassland ecosystems.

## 4. Materials and Methods

### 4.1. Study Sites

**Figure 4:**
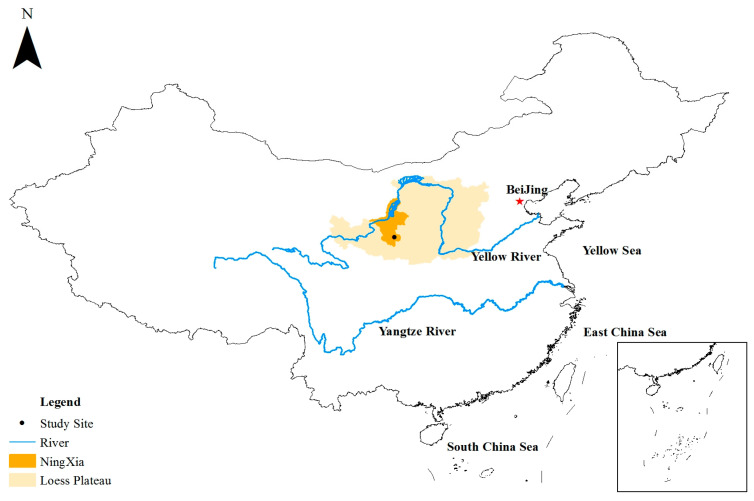
The location of study site.

This study was conducted in the Guyuan Ecological Station of the Institute of Water and Soil Conservation of the Chinese Academy of Sciences, Guyuan City, Ningxia Province, China (shown in Figure 4). This area belongs to the semi-arid ridge hilly area in the west of the Loess Plateau (106°26′ E~106°30′ E, 35°59′ N~36°03′ N) with an altitude ranging from 1534 to 1822 m. This research area experiences a transitional monsoon climate, shifting from semi-arid temperate to warm temperate, with an average annual precipitation of 419.1 mm, an average annual temperature of 6.9 °C, and a dryness of 1.6–2.0. The rainy season predominantly spans from July to October, with August accounting for 24% of the total precipitation. The research area encompasses a total land area of 7.6 km^2^. The soil type was classified as Entisols according to the U.S. Department of Agriculture [52]. Grassland restoration is one of the main forms of ecological restoration in the study area. In order, the dominant species during grassland succession are *Thymus mongolicus* Ronniger (Th. M), *Artemisia sacrorun* Ledeb (Ar. S), and *Stipa bungeana* Trin (St. B). In most cases, the group of St. B and Th. M and the group of St. B and Ar. S are observed in grassland succession.

### 4.2. Litter Collection and Stimulated Field Decomposition Experiments

In this study, sufficient fresh litter from *Stipa bungeana* Trin (St. B), *Artemisia sacrorun* Ledeb, (Ar. S), and *Thymus mongolicus* Celak (Th. M) was collected in September 2018. The litter samples were transferred to the laboratory, where surface impurities were removed using a brush and clean water. Subsequently, the litter was cut into 5 cm pieces and dried in an oven at 65 °C until a constant weight was achieved. A portion of the litter was crushed through a 1 mm sieve for the determination of initial chemical properties. The remaining litter samples were wrapped in kraft paper and stored in a refrigerator.

In this study, we designed seven sets of litter samples for field simulation experiments, including three sets of individual litter and four sets of mixed litter (Table 5). The four mixed litter treatments were created according to the composition of the plant community during grassland succession in this region. Seven treatments were used to simulate the grassland succession in the following order: Th. M, Ar. S, ML1, ML3, ML2, ML4, and St. B. The simulation experiment was conducted on abandoned land and began in May 2019. The soil organic carbon and total nitrogen were 7.57 g/kg and 0.73 g/kg; the soil nitrate nitrogen, ammonium nitrogen, and available phosphorus were 10.92 mg/kg, 5.91 mg/kg, and 5.77 mg/kg; and the concentrations of soil Ca, Mg, Fe, Mn, Zn, and Cu were 6007.47 mg/kg, 1467.96 mg/kg, 3019.84 mg/kg, 605.44 mg/kg, 73.90 mg/kg, and 32.04 mg/kg. Original plant residues on the surface were removed, and the area was divided into 30 × 30 cm grids using PVC partitions. These grids were fenced to prevent accidental damage. Nylon decomposition bags containing 50 g of litter samples were placed in the grids and adhered tightly to the ground. The simulation experiment ended on 11 March 2022. During this period, litter was collected at the 45th day, 90th day, 135th day, 300th day, 345th day, 390th day, 435th day, 480th day, 525th day, 690th day, 735th day, 780th day, 825th day, 870th day, and 1035th day. This study deployed a total of 315 decomposition grids (7 sets of litter samples × 3 duplicates × 15 samplings). The collected samples were taken to the laboratory, where any attachments were removed, followed by weighing, crushing, and then an analysis of chemical properties.

### 4.3. Determination

The determination of the total carbon concentration in the litters was carried out using K_2_Cr_2_O_7_ and concentrated sulfuric acid oxidation methods. The total nitrogen and phosphorus in the litter were measured using H_2_SO_4_-H_2_O_2_ digestion. The total nitrogen content in the digestion solution was determined using a Kjeldahl nitrogen analyzer, and the total phosphorus content was determined using molybdenum yellow colorimetric spectrophotometry. The lignin content was determined using the acidic washing sodium thiosulfate titration method. To determine the concentrations of Ca, Mg, Fe, Mn, Zn, and Cu, 0.1000 g of litter was accurately weighed, mixed with 50 mL of concentrated nitric acid and 1 mL of hydrogen peroxide, and placed in a polytetrafluoroethylene reaction tank, which was then diluted to 50 mL and digested at 200 °C. The concentrations of microelements were measured using an ICP-MS (Agilent 7700, Santa Clara, CA, USA).

### 4.4. Data Analysis

The mass remaining proportion refers to the ratio of the remaining litter mass to the initial mass over a specified decomposition period. A larger residual proportion indicates a slower rate of litter decomposition; conversely, a smaller proportion indicates a faster decomposition rate. The proportion of litter mass retained (MR_t_, %) at a given time t is calculated as follows:

(1) $${\mathrm{MR}}_{t}=\frac{M_{t}}{M_{0}}\times 100\%$$

where M_0_ is the initial mass of litter (g); M_t_ is the mass of litter at time t (g).

The litter decomposition model employed the Olson equation [53], expressed by the following formula:

(2) $$\frac{M_{t}}{M_{0}}=a\;\times \;e^{-kt}$$

where M_0_ is the initial mass of litter (g); M_t_ is the mass of litter at time t (g); a is coefficient factor; and *k* is the decomposition constant.

The accumulation index (AI) is used to represent the accumulation or release of nutrients during the litter decomposition process. When A1 > 100%, it indicates a net accumulation of elements, and when AI < 100%, it indicates a net release of elements. The AI is calculated as follows:

(3) $$\mathrm{AI}=\frac{M_{t}\times X_{t}}{M_{0}\times X_{0}}\times 100\%$$

where X_t_ is the nutrient/element content of litter at time *t* of decomposition (g/kg), and X_0_ is the initial nutrient/element content of litter (g/kg).

To evaluate the non-additive (synergistic or antagonistic) effect (ME) of different litter mixtures on the remaining rate of mass and microelements during decomposition, we measured an observed value (O) and calculated an expected value (E) for each species and mixture. When ME > 0, it represents that the observed remaining rate of mass or microelement is greater than expected, indicating that mixing inhibits litter decomposition or elemental release (antagonistic effect). The ME is calculated as follows:

(4) $$\mathrm{ME}=\frac{O-\;E_{t}}{E}\;\times \;100\%$$

All data in this article were systematically organized using Microsoft Excel 2019 and plotted using Origin 2018. All data points were denoted by the standard deviation of the mean. A one-way ANOVA and a minimum significance test, conducted using IBM SPSS Statistics 25, were employed to compute the significant differences in the residual proportion, macro- and microelement contents, and lignin content during the decomposition process across different litter groups. The correlation between the decomposition characteristics of the litter and its chemical properties was determined using Pearson correlation analysis.

## 5. Conclusions

In this study, we conducted long-term (1035 days) grass litter decomposition experiments to simulate the decomposition of litter of different plant compositions of grassland succession in the Loess Plateau, China. Our findings showed that mixed litter enhanced the decomposition rate compared to single litter. The fast–fast decomposing litter (St. B and Ar. S), with the same proportion, had a faster decomposition rate and lower mass remaining rate compared to the fast–slow decomposing litter (St. B and Th. M), indicating that the positive grassland succession effectively accelerates the decomposition of litter in the Loess Plateau. We also observed that the concentration and amount of Ca, Mg, Fe, Mn, Zn, and Cu exhibited a dynamic behavior pattern of short-term increase and long-term decay in both individual and mixed litters. Compared to the individual St. B litter, the litter mixture inhibited Ca release and promoted Mg, Cu, and Zn release, revealing that the process of grassland succession may modify the release characteristics of different microelements following litter decomposition. The dynamic behaviors of microelements deserve attention in future studies due to their different release characteristics. Furthermore, we found nitrogen and microelements to periodically co-drive the decomposition of typical grass litter in the Loess Plateau by employing stepwise regression analysis. In summary, these findings helped improve our understanding of grassland restoration in the Loess Plateau region, the dynamic behavior model of element turnover in the process, and the mechanisms regulating the litter decomposition system.

## Figures and Tables

**Table 1:** The exponential relationship between the residual proportion and cultivation time.

Litter Groups	Regression Equation	The Decomposition Constant (*k*)	*R* ^2^	*p*
St. B	$\frac{Mt}{M0}$ = 0.9506 × e^−0.0209t^	0.0209	0.9512	<0.0001
Ar. S	$\frac{Mt}{M0}$ = 0.8363 × e^−0.0207t^	0.0207	0.8576	<0.0001
Th. M	$\frac{Mt}{M0}$ = 0.9469 × e^−0.0133t^	0.0133	0.9437	<0.0001
ML1	$\frac{Mt}{M0}$ = 0.9489 × e^−0.0229t^	0.0229	0.9813	<0.0001
ML2	$\frac{Mt}{M0}$ = 0.8772 × e^−0.0242t^	0.0242	0.9391	<0.0001
ML3	$\frac{Mt}{M0}$ = 0.8747 × e^−0.0224t^	0.0224	0.9644	<0.0001
ML4	$\frac{Mt}{M0}$ = 0.8367 × e^−0.0254t^	0.0254	0.8956	<0.0001

Note: the decomposition constant (*k*) was calculated by fitting the litter mass remaining ($\frac{Mt}{M0}$) and decomposition time (t) using the Olson equation.

**Table 2:** The mixing effects of litter on the mass remaining rate and accumulation index (AI) of microelements during litter decomposition.

Time	Mass Remaining Proportion				Ca−AI				Mg−AI				Fe−AI				Mn−AI				Cu−AI				Zn−AI			
(d)	ML1	ML2	ML3	ML4	ML1	ML2	ML3	ML4	ML1	ML2	ML3	ML4	ML1	ML2	ML3	ML4	ML1	ML2	ML3	ML4	ML1	ML2	ML3	ML4	ML1	ML2	ML3	ML4
0	2.79	−0.25	−9.58	−12.34	39.57	26.26	9.14	23.79	2.89	34.65	−18.76	7.29	97.13	88.28	23.23	31.81	68.26	42.88	16.98	39.78	9.44	−6.91	−18.40	−26.94	−9.82	−11.08	−33.59	−31.62
45	0.35	−2.30	−10.52	−17.64	17.93	34.72	4.45	34.80	−9.01	6.75	−23.45	−2.68	38.87	32.20	14.43	18.71	42.45	31.85	15.13	39.73	−51.31	−55.87	−56.50	−62.21	−25.24	−21.15	−24.96	−24.00
90	−0.72	−3.92	−8.51	−19.23	82.92	33.25	43.22	67.47	0.28	−16.69	−26.21	−4.85	38.52	26.59	3.72	2.04	29.86	2.92	2.29	21.59	12.61	−37.24	−4.69	−36.61	−8.95	−26.11	−10.39	−28.91
135	−7.14	−10.28	−14.78	−22.11	136.22	188.44	196.31	254.28	−2.59	67.41	−6.70	30.23	102.69	340.00	132.39	161.13	10.93	59.03	9.03	35.56	16.31	15.25	9.55	−31.00	−7.68	0.31	−36.04	−26.63
300	−7.63	−7.09	−13.11	−21.57	−17.02	−25.61	5.42	6.14	−83.93	−70.56	−82.29	−79.73	18.36	76.69	29.27	31.23	9.77	11.69	4.01	17.15	−42.18	−59.00	−77.96	−75.41	−65.59	−60.70	−74.34	−73.32
345	−11.21	−7.77	−17.61	−22.40	−31.16	−28.35	−39.99	−31.50	−40.55	−38.85	−78.39	−53.52	7.22	55.07	0.69	1.15	8.25	18.25	−7.29	5.77	−15.39	16.57	−4.56	−48.90	−3.49	−15.77	−21.89	−25.25
390	−9.65	−10.75	−16.89	−22.46	86.64	70.72	114.44	107.78	−50.76	−7.72	−62.58	−28.03	−21.02	21.75	19.48	15.63	−32.57	16.60	−6.64	5.01	−67.62	−65.44	−65.61	−79.38	−18.11	−25.85	11.37	−8.28
435	−13.90	−12.65	−15.96	−22.06	22.67	46.97	51.51	54.50	−28.93	−32.04	−50.62	−3.33	−20.43	36.84	−18.06	−21.97	−3.27	18.76	−9.77	−0.13	−17.65	−50.99	−30.39	−46.60	−20.48	−31.68	−25.71	−27.56
480	−14.92	−14.65	−16.88	−25.78	35.77	41.13	54.08	55.16	−49.55	−33.85	−48.70	−19.88	−19.24	32.78	−17.88	−27.25	−3.91	13.20	−9.82	−9.48	−19.96	−51.47	−34.04	−53.62	−18.18	−34.12	−28.88	−29.76
525	−13.28	−6.69	−7.04	−20.75	45.95	56.69	72.16	56.66	−48.50	−32.16	−36.73	−16.03	−11.78	47.03	2.87	−16.54	−3.41	20.25	1.04	−1.21	−20.26	−46.03	−24.44	−50.77	−18.15	−12.34	−22.28	−35.58
690	−11.46	−6.82	−10.68	−22.84	70.47	74.74	65.41	57.62	−51.46	−40.75	−54.60	−12.20	−24.86	29.39	−33.49	−23.40	6.84	12.25	−23.97	−28.26	2.38	−22.73	−10.38	−41.60	−9.65	−14.39	−17.51	−47.84
735	−12.85	−4.12	−10.30	−21.53	49.01	47.62	77.18	63.43	−48.39	−59.64	−61.62	−38.91	−15.44	2.81	−24.25	−24.90	−5.66	−14.52	−35.25	−25.76	−6.62	−33.73	−18.94	−39.46	−19.05	−6.73	−18.72	−48.39
780	−12.36	−3.23	−15.77	−24.64	31.63	46.87	19.18	92.60	−11.13	−3.10	−43.77	21.49	34.15	121.10	17.58	43.53	20.82	19.70	2.19	15.20	47.22	−28.92	22.44	−39.85	−14.41	−16.64	−19.40	−35.53
825	−11.47	−13.34	−16.19	−25.39	26.80	11.84	50.24	48.27	−31.42	−32.90	−58.40	−8.99	−27.84	−4.76	−38.73	−36.31	−17.94	−16.31	−27.85	−11.24	−21.32	1.25	−8.12	−47.38	−26.99	−23.90	−20.75	−35.80
870	−9.97	−10.94	−17.79	−24.05	4.08	18.60	20.47	10.39	−14.45	−4.05	−37.14	−20.68	−30.73	19.90	−23.71	−17.39	−3.28	10.53	−36.53	−20.29	−31.74	−7.21	−35.35	−51.37	−18.34	−19.68	−23.71	−43.35
1035	2.79	−0.25	−9.58	−12.34	39.57	26.26	9.14	23.79	2.89	34.65	−18.76	7.29	97.13	88.28	23.23	31.81	68.26	42.88	16.98	39.78	9.44	−6.91	−18.40	−26.94	−9.82	−11.08	−33.59	−31.62

**Table 3:** The correlation relationships between initial litter chemical properties (concentration variables) and mass remaining rate at 1035th day.

Correlations	TN	Lignin	Ca	Mg	Fe	Mn	Zn	Cu
Mass remaining rate	−0.778 *	0.756 *	0.674	0.790 *	0.821 *	0.873 *	0.229	0.349

Note: * indicate that the relationships were significant at 0.05 statistical levels, respectively.

**Table 4:** Stepwise regression to examine the stage factors modulating the decomposition processes of single and mixed litter.

Variables	TN	Lignin	Ca	Mg	Fe	Mn	Zn	Cu	Constant	*p*	R^2^
MR_single (0–525)_	/	/	−0.011 (100%)	/	/	/	/	/	0.910	0.002	0.299
MR_single (690–1035)_	−0.018 (53.07%)	/	/	/	/	−0.001 (23.84%)	/	0.007 (23.09%)	0.951	0.027	0.807
MR_mixed (0–525)_	/	/	−0.016 (18.37%)	0.126 (43.39%)	/	−0.002 (38.24%)	/	/	1.001	0.000	0.700
MR_mixed (0–525)_	−0.010 (100%)	/		/	/	/	/	/	0.637	0.016	0.238

Note: MR_single (0–525)_ and MR_single (690–1035)_ indicate the mass remaining rate of single litters at 0–525 days and 690–1035 days, respectively. Percentages in brackets indicate the explanation degree of the dependent variable by the independent variable.

**Table 5:** The litter composition of each treatment.

Treatments	Litter Composition (50 g)
St. B	St. B litter (100%)
Ar. S	Ar. S litter (100%)
Th. M	Th. M litter (100%)
ML1	St. B litter (55%) + Th. M litter (45%)
ML2	St. B litter (55%) + Ar. S litter (45%)
ML3	St. B litter (75%) + Th. M litter (25%)
ML4	St. B litter (75%) + Ar. S litter (25%)

## Data Availability

Data are available from the authors on reasonable request.

## Supplementary Materials

The following supporting information can be downloaded at: https://www.mdpi.com/article/10.3390/plants13060753/s1, Figure S1. The liner regressions between lignin accumulation index and Ca concentration (a) and Mn concentration (b) of mixed litter sets at 0–525 days. Figure S2. The liner regressions between the litter mass remaining rate and lignin accumulation index of single sets (a) and mixed sets (b) at decomposing 0–525 days (green dots) and 690–1035 days (blue dots). Figure S3. The variations of concentration and accumulation index of TN (a1 and a2) and lignin (b1 and b2) during the litter decomposition. Table S1. The initial chemical property of leaf litters.
