# Supplementary material for: Nitrogen and Microelements Co-Drive the Decomposition of Typical Grass Litter in the Loess Plateau, China
Source: Plants (Basel). 2024 Mar 7;13(6):753. doi: 10.3390/plants13060753 (PMC10975666; doi:10.3390/plants13060753)
Supplement: Supplementary file 1 [file plants-13-00753-s001.zip › plants-2853294-supplementary.pdf]

plants

Article

Nitrogen and Microelements Co-Drive the Decomposition of Typical Grass Litter in the Loess Plateau, China

Yun Xiang [1,2], Haoning Chen [2], Weiqi Feng [2], Yongli Wen [2], Ying Xie [3], Man Cheng [2,*] and Hua Li [2,*]

[1] College of Resources and Environment, Shanxi Agricultural University, Jinzhong 030801, China; xiangyun@sxau.edu.cn
[2] Institute of Loess Plateau, School of Environmental & Resource Sciences, Shanxi University, Taiyuan 030006, China; chn13753900846@163.com (H.C.); 202323202006@email.sxu.edu.cn (W.F.); ylwen@sxu.edu.cn (Y.W.)
[3] Shanxi Dadi Minji Ecological Environment Company Limited, Taiyuan 030002, China; xieying19926@163.com
* Correspondence: chengman@sxu.edu.cn (M.C.); lihua@sxu.edu.cn (H.L.); Tel.: +86-139-3460-3466 (H.L.)

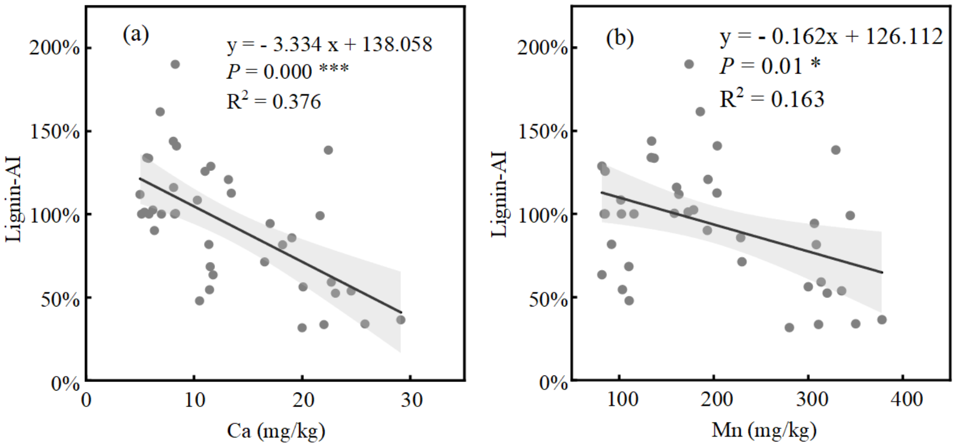

Figure S1. The liner regressions between lignin accumulation index and Ca concentration (a) and Mn concentration (b) of mixed litter sets at 0-525 days.

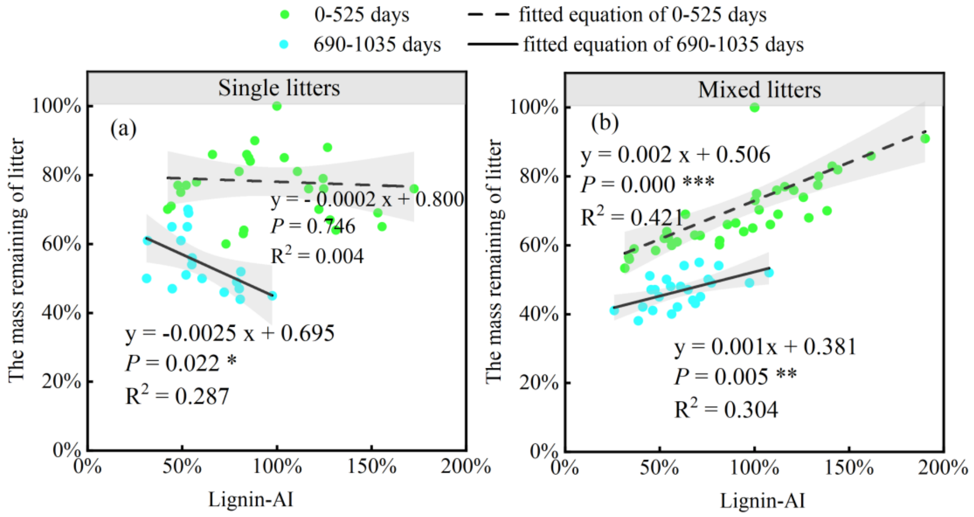

Figure S2. The liner regressions between the litter mass remaining rate and lignin accumulation index of single sets (a) and mixed sets (b) at decomposing 0-525 days (green dots) and 690-1035 days (blue dots).

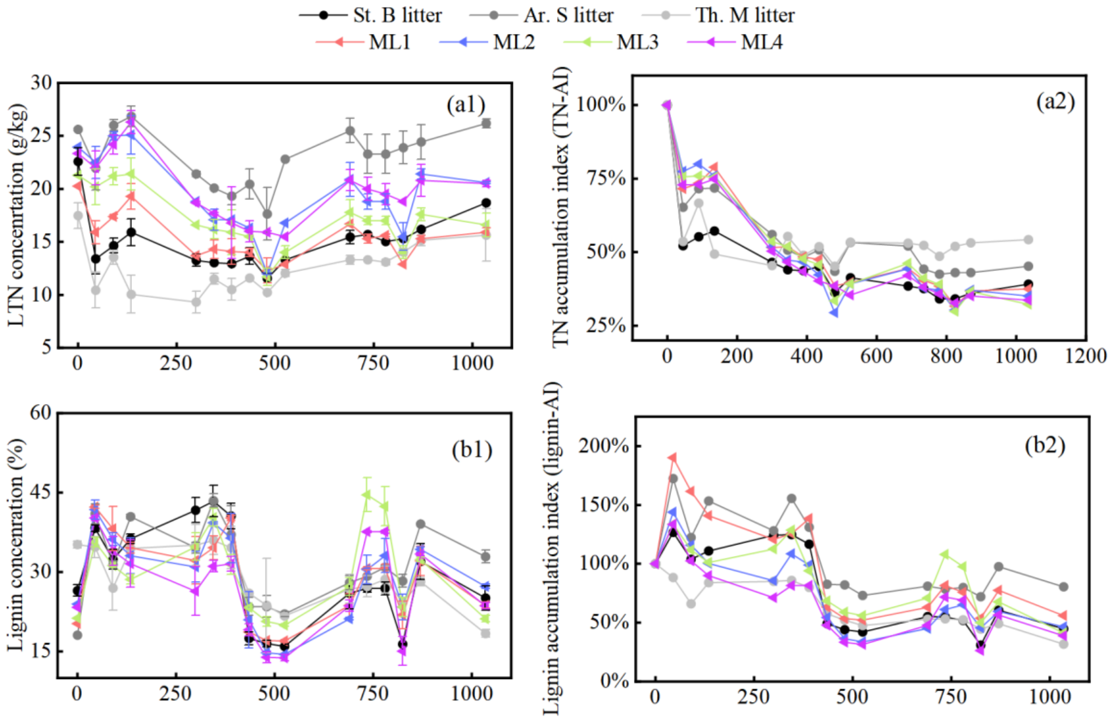

Figure S3. The variations of concentration and accumulation index of TN (a1 and a2) and lignin (b1 and b2) during the litter decomposition.

Table S1. The initial chemical property of leaf litters.

**Chemical variables**	**St. B**	**Ar. S**	**Th. M**
TC (g/kg)	452.0±5.2 b	534.0±19.2 a	462.8±11.7 b
TN (g/kg)	22.6±1.3 b	25.6±0.6 a	17.5±1.2 c
TP (g/kg)	1.9±0.1 b	2.4±0.1 a	1.8±0.2 b
Lignin (%)	26.5±1.1 b	18.1±0.6 c	35.2±0.7 a
C/N	20.0±0.9 a	20.8±8.3 a	26.5±2.5 a
C/P	240.7±3.8 a	219.0±2.6 a	263.6±34.5 a
N/P	12.0±0.3 a	10.5±2.7 b	9.9±2.2 b
Lignin/N	11.8±0.9 b	7.1±9.0 b	20.1±3.0 a
Ca (mg/kg)	686.8±107.0c	1201.9±30.0b	1477.0±141.0a
Mg (mg/kg)	96.9±18.0c	265.1±32.0b	403.6±16.0a
Fe (mg/kg)	125.7±3.3b	174.3±1.8b	373.4±3.5a
Mn (mg/kg)	86.1±5.6b	82.3±5.0b	151.8±4.3a
Cu (mg/kg)	8.6±0.5c	35.1±0.4a	25.0±1.0b
Zn (mg/kg)	23.9±1.6b	47.0±2.5a	41.2±0.9a

Note: Different lowercase letters indicate significant differences among different litter species.
